# Successful Systemic Steroid Administration for the Treatment of Edematous Anastomotic Stenosis After the Laparoscopic Augmented Rectangle Technique for Billroth I Reconstruction for Laparoscopic Distal Gastrectomy

**DOI:** 10.7759/cureus.40914

**Published:** 2023-06-25

**Authors:** Tomoaki Ito, Mutsumi Sakurada, Tomoyuki Kushida, Kenichiro Tanaka, Koichi Sato

**Affiliations:** 1 Department of Surgery, Juntendo University Shizuoka Hospital, Juntendo University School of Medicine, Shizuoka, JPN

**Keywords:** systemic steroid, intracorporeal anastomosis, edematous anastomotic stenosis, billroth i reconstruction, augmented rectangle technique

## Abstract

Edematous anastomotic stenosis is a well-known complication following Billroth I anastomosis for distal gastrectomy. Currently, there is no established treatment for this condition. A 54-year-old female patient underwent the augmented rectangle technique for Billroth I reconstruction after total laparoscopic distal gastrectomy for early gastric cancer. On postoperative day (POD) 9, the patient started vomiting. During the conservative waiting period, edematous anastomotic stenosis was diagnosed using imaging on PODs 11 and 13. Systemic steroid administration was initiated on POD 13, and the drainage volume of the nasogastric tube decreased four days after initiation. The edematous anastomosis stenosis improved, and gastrografin flowed into the duodenum on POD 19. Food intake was started on POD 20. Oral steroid administration was continued after hospital discharge and gradually terminated. Systemic steroid treatment may help improve edematous anastomotic stenosis.

## Introduction

Billroth I anastomosis is a common reconstruction procedure performed after distal gastrectomy for gastric cancer. Several intracorporeal anastomosis procedures, including delta-shaped anastomosis, [1] the triangulating stapling technique [2], and the augmented rectangle technique (ART), which was developed and reported by Fukunaga et al. in 2018 [3], have been considered for laparoscopic distal gastrectomy. Despite these advancements, anastomotic stenosis is associated with a complication rate of 0% to 3.2% [2-4]. The treatment for anastomotic stenosis is endoscopic balloon dilatation or conservative waiting until natural recovery occurs, which are often ineffective; therefore, there is no established treatment for this condition.

In 2018, at our institution, we adopted ART for Billroth I anastomosis after laparoscopic distal gastrectomy. We report systemic steroid administration for a case involving edematous anastomotic stenosis treated using the ART after laparoscopic distal gastrectomy.

## Case presentation

A 54-year-old female patient was referred to the authors’ hospital. She had early-stage gastric cancer in the lower third of the stomach. She underwent the ART for laparoscopic distal gastrectomy with Billroth I anastomosis [3].

The reconstruction procedure was performed as follows. After lymph node dissection, the duodenum and stomach were cut using a 60-mm endoscopic linear stapler (ELS). Next, the entry holes created for the insertion of the ELS were opened at the resection stump on the greater curvature of the duodenum and stomach. Subsequently, the 60-mm ELS was fired in the posterior walls of the stomach and duodenum. Then, the entry hole was closed using a 45-mm ELS, followed by the use of a 60-mm ELS. The shape of the anastomosis was an augmented rectangle. The mean operative time was 327 min, and the mean blood loss was 25 g.

Oral intake was initiated on postoperative day (POD) 4, and it was continued until POD 8. However, the patient started vomiting on POD 9. She did not experience fever or abdominal pain. Laboratory examination results revealed an increased C-reactive protein level of 11.32 mg/dL and a normal white blood cell count of 6,900/μL on POD 11 (Table 1).

**Table 1:** Blood test results on POD 11 POD, postoperative day; WBC, white blood cell count; RBC, red blood cell count; Hb, hemoglobin; Hct, hematocrit; MCV, mean corpuscular volume; MCH, mean corpuscular hemoglobin; MCHC, mean corpuscular hemoglobin concentration; Plt, platelet; ALB, albumin; T-Bil, total bilirubin; AST, aspartate aminotransferase; ALT, alanine aminotransferase; LDH, lactate dehydrogenase; CPK, creatine phosphokinase; Amy, amylase; GLU, glucose; BUN, blood urea nitrogen; Cre, creatinine; CRP, C-reactive protein.

Test	Unit	Reference range	Value
WBC	10^9^/L	3.6-8.9	6.9
RBC	10^12^/L	3.80-5.04	4.03
Hb	g/dL	11.1-15.2	12
Hct	%	35.6-45.4	35.7
MCV	fL	84.2-99.0	88.6
MCH	pg	27.2-33.0	29.8
MCHC	%	31.8-34.8	33.6
Plt	10^9^/L	153-346	340
ALB	g/dL	4.0-5.2	3.3
T-Bil	mg/dL	0.4-1.2	0.5
AST	IU/L	5-37	14
ALT	IU/L	6-43	14
LDH	IU/L	119-221	145
CPK	IU/L	47-200	21
Amy	IU/L	43-124	42
GLU	mg/dL	65-109	132
BUN	mg/dL	8-20	7.4
Cre	mg/dL	0.5-0.8	0.4
Sodium	mEq/L	135-145	137
Potassium	mEq/L	3.5-5.0	4.1
Chloride	mEq/L	96-107	100
CRP	mg/dL	0-0.30	10.32

**Figure 1:**
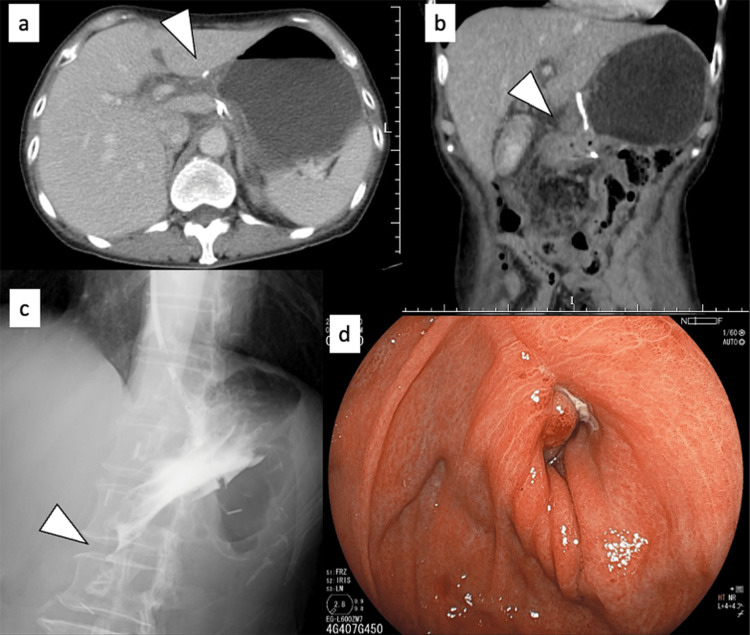
Images obtained before systemic steroid administration (between POD 11 and POD 13). The axial CT scan (a), coronal CT scan (b), and fluoroscopic examination (c) show that the gastroduodenal anastomosis exhibits edematous stenosis, and the remnant stomach is dilated because there is no gastric juice flow. The endoscopy (d) revealed that the duodenal mucosa is edematous and protruded into the lumen of the anastomosis. Arrowhead indicates the anastomosis. POD, postoperative day.

A nasogastric tube was placed to drain the residual gastric contents on POD 11. Abdominal computed tomography (CT) performed on POD 11 revealed no leakage, gastric food stasis, or edematous anastomosis (Figure 1 a and b). During the conservative waiting period, a fluoroscopic examination on POD 13 revealed that the gastrografin could not flow into the duodenum (Figure 1c). Endoscopy revealed that the duodenal mucosa was edematous and protruded into the lumen of the anastomosis (Figure 1d). We consulted with the patient and explained that the treatment options for edematous anastomotic stenosis were conservative waiting, endoscopic balloon dilatation, and systemic steroid administration, that there is no evidence of the effectiveness of any of those treatments, and that there have been few reports of the effectiveness of steroid treatment. We also explained the possibility of a more extended hospital stay if conservative waiting and endoscopic balloon dilatation were ineffective. After acknowledging the possible side effects of steroids, the patient chose systemic steroid administration to shorten the hospital stay.

**Figure 2:**
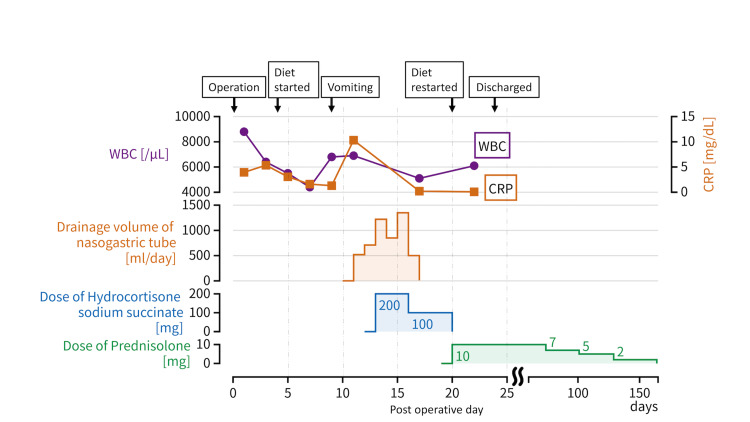
Clinical treatment course. The drainage volume of the nasogastric tube decreased soon after the initiation of systemic steroids. CRP, C-reactive protein; WBC, white blood cell count.

On POD 13, systemic steroids were started. Hydrocortisone sodium succinate (200 mg/day) was administered for three days, followed by 100 mg/day for four days. The patient’s clinical course is shown in Figure 2.

**Figure 3:**
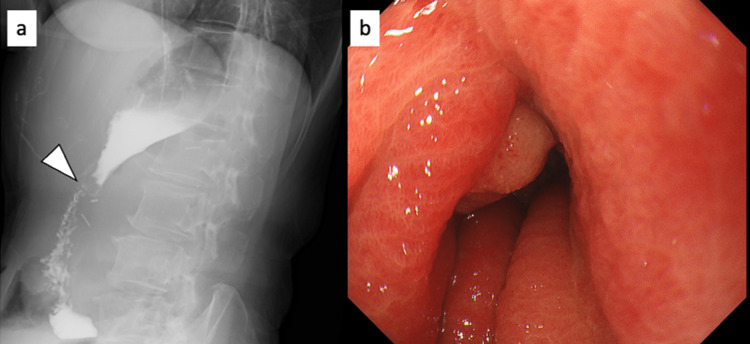
Images obtained after systemic steroid administration (POD 18 and POD 19). The endoscopic examination revealed improvement of the edematous anastomotic stenosis (b) and the flow of gastrografin into the duodenum (a). The arrowhead indicates anastomosis. POD, postoperative day.

The drainage volume of the nasogastric tube decreased after the initiation of systemic steroid administration. A fluoroscopic examination on POD 18 revealed that gastrografin could flow into the duodenum (Figure 3a). The nasogastric tube was removed on POD 18. The endoscopic study performed on POD 19 showed that the edematous mucosa of the anastomosis had improved (Figure 3b). Therefore, oral intake was initiated on POD 20. She was administered 10 mg/day of prednisolone on POD 20. On POD 24, the patient was discharged from the hospital. After discharge from the hospital, the prednisolone dose was slowly tapered over a long period of time because she could not frequently attend outpatient clinic appointments. Steroid administration should have been completed earlier, but it was ultimately terminated on POD 164.

## Discussion

From inception to February 2023, we searched the PubMed database for case reports using the following terms: “Anastomotic stenosis” AND “Steroid” AND “Gastrectomy”. Only one case [5] was reported. Furthermore, from inception to February 2023, we also searched a Japanese medical bibliographic database, the Igaku Chuo Zasshi Database (ICHUSHI), for Japanese-language case reports using the following terms in Japanese: “Anastomotic stenosis” AND “Steroid” AND “Gastrectomy”. Three cases were reported. In PubMed, our current case is the second reported case involving the effective administration of systemic steroids for edematous anastomotic stenosis after distal gastrectomy.

Including this case, we have summarized a total of five such cases; therefore, the cases of two male and three female patients were included in this study (Table 2) [5-8]. The median age of these patients was 76 years (range, 54-79 years). Four of these five patients underwent laparoscopic distal gastrectomy followed by Billroth I anastomosis with a stapler. The remaining patient underwent open distal gastrectomy followed by Billroth II anastomosis with hand-sewn sutures [6]. Symptom onset occurred on POD 9 (range, POD 5-22). Three of these patients experienced vomiting. Two of these patients experienced abdominal pain.

**Table 2:** Summary of edematous anastomotic stenosis cases treated with systemic steroids POD, postoperative day; UGI, upper gastrointestinal tract investigation.

Case number	Author	Year	Sex	Age (years)	Reconstruction type	Anastomosis method	Steroid type	Initial steroid dosage (mg/day)	Symptom onset (POD)	Symptoms	Initiation of systemic steroids (POD)	Effects observed (POD)	Details
1	Ohashi et al. [6]	2013	Female	79	Billroth II	Hand-sewn	Prednisolone	30	8	Vomiting	97	110	Improvement of flow during the UGI
2	Fujihata et al. [7]	2017	Female	79	Billroth I	Stapler	Hydrocortisone sodium succinate	200	5	Vomiting	21	22	Improvement of flow during the UGI
3	Arima et al. [5]	2020	Male	72	Billroth I	Stapler	Prednisolone	40	9	Left abdominal pain	56	57	Improvement of flow during the UGI
4	Takahara et al. [8]	2022	Male	76	Billroth I	Stapler	Hydrocortisone sodium succinate	100	22	Epigastralgia	28	30	Improvement of flow during the UGI
5	Our case		Female	54	Billroth I	Stapler	Hydrocortisone sodium succinate	200	9	Vomiting	13	18	Improvement of flow during the UGI

Arima et al. [5] reported multiple treatments for anastomotic stenosis before the initiation of systemic steroid treatment. Endoscopic balloon dilatation and local steroid injections into the stenosis were inadequate treatments. Subsequently, they administered systemic steroids to their patients. Surprisingly, the effectiveness of systemic steroids was observed in one patient the day after their initiation. The other patients also showed improved passage during the upper gastrointestinal investigation within 14 days of the initiation of systemic steroid administration. Shoji et al. reported that systemic steroid treatment for stenosis after endoscopic submucosal resection was more effective than local steroid injections [9]. Regarding the current case, we did not perform endoscopic balloon dilatation or local steroid injection because the patient requested systemic steroid administration. Therefore, systemic steroids were promptly initiated for edematous anastomotic stenosis and immediately produced an effect.

A delta-shaped anastomosis was performed for three of our five patients [1]. Billroth I anastomosis using a delta-shaped anastomosis is one of the most common reconstruction methods for such cases. We performed Billroth I anastomosis using the ART to create an entirely intracorporeal anastomosis for the current case. Billroth I anastomosis using the ART could create a wider anastomosis than the delta-shaped anastomosis [3]. However, Nishiguchi et al. reported that the incidence of anastomotic stenosis using the ART method was 2.6% [4]. For our current case, although the cause of the edematous anastomotic stenosis was unknown, the inflammatory findings, elevated C-reactive protein levels, and white blood cell count suggested that the cause was ischemia or inflammation of the surrounding anastomosis.

Steroids have many beneficial effects, including anti-inflammatory effects, collagen degradation, and edema [10]. These effects could help improve edematous anastomotic stenosis in our current case. No consensus regarding the type or dosage of steroids has been reached. Therefore, we initially used hydrocortisone sodium succinate for our current case (this was also used for two of our other cases). Prednisolone therapy was used for the remaining cases. However, the equivalent dosage was almost the same for all cases. Therefore, the initial dosage of systemic steroids for the current case may have been sufficient. The tapering period for the oral steroid dosage for this case was long. However, because of the potential side effects, a shorter tapering period for the steroid dosage is desirable. Because the dosage and duration of oral steroids after discharge varied, there is no consensus at this time.

## Conclusions

In conclusion, this case report highlights the successful use of systemic steroid administration for the treatment of edematous anastomotic stenosis after laparoscopic distal gastrectomy with the ART for Billroth I anastomosis. The ability of systemic steroids to improve the patient’s condition was evident in this case; both prompt resolution of symptoms and improved passage were observed within a relatively short period. This case report adds to the limited existing evidence of the potential benefits of systemic steroids for edematous anastomotic stenosis in selected cases. Further research is necessary before standardized guidelines for steroid administration can be established.
